# Treatment recommendations made by a consultant psychiatrist to improve the quality of care in a collaborative mental health intervention in rural Nepal

**DOI:** 10.1186/s12888-020-2464-1

**Published:** 2020-02-05

**Authors:** Pragya Rimal, Duncan Maru, Lydia Chwastiak, Pawan Agrawal, Deepa Rao, Sikhar Swar, David Citrin, Bibhav Acharya

**Affiliations:** 1 Nyaya Health Nepal, Kathmandu, Nepal; 2 Departments of Global Health System Design and Global Health, Internal Medicine, and Pediatrics, Mount Sinai School of Medicine, New York, NY USA; 3 Arnhold Institute for Global Health, Mount Sinai School of Medicine, New York, NY USA; 4 Department of Psychiatry & Behavioral Sciences, University of Washington, Seattle, WA USA; 5 Department of Global Health, University of Washington, Seattle, WA USA; 6 Northwest Mental Health Technology Transfer Center, University of Washington, Seattle, WA USA; 7 Department of Global Health, University of Washington, Seattle, WA USA; 8 Department of Psychiatry and Behavioral Sciences, University of Washington, Seattle, WA USA; 9 Department of Psychiatry, Kathmandu Medical College, Duwakot, Nepal; 10 Department of Anthropology, University of Washington, Seattle, WA USA; 11 Department of Global Health, University of Washington, Seattle, WA USA; 12 Henry M. Jackson School of International Studies, University of Washington, Seattle, WA USA; 13 Department of Psychiatry, University of California, San Francisco, CA USA

**Keywords:** Mental health, LMIC, Global mental health, Nepal

## Abstract

**Background:**

The Collaborative Care Model (CoCM) for mental healthcare, where a consulting psychiatrist supports primary care and behavioral health workers, has the potential to address the large unmet burden of mental illness worldwide. A core component of this model is that the psychiatrist reviews treatment plans for a panel of patients and provides specific clinical recommendations to improve the quality of care. Very few studies have reported data on such recommendations. This study reviews and classifies the recommendations made by consulting psychiatrists in a rural primary care clinic in Nepal.

**Methods:**

A chart review was conducted for all patients whose cases were reviewed by the treatment team from January to June 2017, after CoCM had been operational for 6 months. Free text of the recommendations were extracted and two coders analyzed the data using an inductive approach to group and categorize recommendations until the coders achieved consensus. Cumulative frequency of the recommendations are tabulated and discussed in the context of an adapted CoCM in rural Nepal.

**Results:**

The clinical team discussed 1174 patient encounters (1162 unique patients) during panel reviews throughout the study period. The consultant psychiatrist made 214 recommendations for 192 (16%) patients. The most common recommendations were to revisit the primary mental health diagnosis (16%, *n* = 34), add or increase focus on counselling and psychosocial support (9%, *n* = 20), increase the antidepressant dose (9%, n = 20), and discontinue inappropriate medications (6%, *n* = 12).

**Conclusions:**

In this CoCM study, the majority of treatment plans did not require significant change. The recommendations highlight the challenge that non-specialists face in making an accurate mental health diagnosis, the relative neglect of non-pharmacological interventions, and the risk of inappropriate medications. These results can inform interventions to better support non-specialists in rural areas

## Background

A substantial shortage of mental healthcare providers in low- and middle-income countries (LMICs) [1] has left 75–80% of patients without even basic mental healthcare [2]. In Nepal, there are about 100 psychiatrists, most of them concentrated in urban areas, [3]. In such settings, a common strategy to expand access to mental healthcare is by using task-sharing, where non-specialists such as primary care providers (PCPs), deliver mental healthcare [4–6]. Various interventions that integrate mental health services into primary care delivery have been implemented in LMICs [7]. The Collaborative Care Model (CoCM) is a specific team-based approach to task-sharing that incorporates non-specialist PCPs, care managers, and consultant psychiatrists to deliver mental health services [8]. In CoCM, the PCPs conduct initial screening and dispense medications, the care managers conduct an in-depth psychosocial assessment, provide counselling and coordinate care, while the whole team reviews the treatment plans for all the patients during a panel review with a consultant psychiatrist [9]. Over 80 randomized controlled trials around the world have found that CoCM is effective in improving mental health outcomes, and that the panel review between the psychiatrist and the primary care team is a crucial component of the intervention [10, 11].

Because medical education curricula in LMICs often have limited coverage of mental health [12], it is critical to train and continually support the PCPs in evidence-based assessment and treatment for mental illness. A crucial component of CoCM is the panel review where all team members (the PCPs, the counselor/care manager, and the psychiatrist) meet and review the presentation, diagnosis, treatment plan, and response to treatment for a panel of patients. These panel reviews result in recommendations made by the psychiatrist, assisting in evidence-based treatment to improve the quality of care. Despite the critical importance of the information exchanged during panel reviews, very little has been published about common recommendations made by the psychiatrist. Such data can be helpful in developing training programs, incorporating appropriate decision-support tools, anticipating errors and other challenges, and for continuous quality improvement. To our knowledge, the contents and processes of such panel reviews have not been reported in the literature. Here, we describe the process and results from codifying such recommendations to uncover common gaps in care at a primary care clinic in rural Nepal.

## Methods

### Site and intervention

The study was conducted in the primary care outpatient clinic of Bayalpata Hospital, a district-level facility operated by *Possible*, a non-profit organization, in close partnership with the government of Nepal since 2008 [13]. The clinic is located in Achham, one of the poorest districts in Nepal that was severely affected by the 10-year Maoist War that ended in 2006 [14]. The hospital is a referral center for the region and serves a catchment area of 60,000 people. Every day, approximately 300–400 patients visit the clinic which is staffed by 20 PCPs.

The CoCM at our study site integrates PCPs, counselors and an offsite psychiatrist. PCPs are clinicians with varying degree of training including health assistants with 36 months of training and MBBS doctors with Bachelor of Medicine, Bachelor of Surgery who complete 5 years of training and a year of clinical rotation. Their medical training included minimal, if any mental health topics [15]. Counselors are a special cadre of mental health professionals who are trained to obtain mental health history, understand psychosocial problems and provide support through skills like counselling, psycho-education and relaxation techniques [16]. Some counselors may also have completed medical training so they can work at the interface of primary care. In our study, some counselors have had prior medical training (ranging from 18 to 36 months) and all received 6 months of behavioral health training. The consultant psychiatrist provides remote supervision by reviewing the panel of patients of all new and high priority follow up cases- with the counselors to ensure the treatment is of high quality and provides on-site training once every quarter [9].

CoCM was introduced in June 2016 with training, communication tools, and workflows refined over the subsequent 6 months. In this care model PCPs evaluate patients, rule out non-psychiatric conditions and upon suspicion of mental illness, direct the patients to same-day visit with counselors located in adjacent offices. They conduct a full psychosocial evaluation, including administration of standardized scales, such as the adapted and validated patient health questionnaire (PHQ-9) [17], and assist PCPs in making the mental health diagnosis and developing a treatment plan. After the counselors’ evaluation, patients return to the PCP for diagnosis and medication prescription (if indicated) and are scheduled for follow-up. Each week, the counselors review their patient panels (discuss the presenting symptoms, results from the standardized scales, mental health diagnosis, and treatment plan) with the consultant psychiatrist over telephone or video conferences*.* During the panel review, the psychiatrist and the local clinical team ensure that the recommendations are feasible. This is accomplished in several ways: a) the local team provides immediate feedback during the panel review if the psychiatrist’s recommendations cannot be followed, so that the psychiatrist can make adjustments; b) the psychiatrist makes quarterly visits to the sites to maintain awareness of the local capacities and limitations; c) the panel review also includes discussions of follow-up patients who are not improving, and if previous recommendations were not feasible, they are modified; and d) during the quarterly visits, the psychiatrist provides hands-on training to further capacitate providers to continually improve the range of interventions that are feasible*.* These panel reviews last two to 4 h and all new and high-priority follow-up patients are discussed. Counselors document the recommendations made by the psychiatrist in *Possible*’s electronic health record (EHR) system [18]. When patients return for follow-up, these recommendations pop-up in the EHR, prompting PCPs to implement them.

### Data sources

All data for this study were extracted from *Possible’*s integrated EHR platform (nepalehr.org). Counselors document recommendations from the psychiatrist during panel reviews as free text directly in the EHR. In addition, demographic information is collected at registration and clinical diagnosis, is entered by clinicians during a patient encounter.

### Data analysis

We performed inductive content analysis on the recommendations, provided as free text and documented by the counselors. Each recommendation statement was coded with a specific theme. e.g., a statement such as *“increase fluoxetine to 40mg daily” was coded under “increase antidepressant dose”.* Two coders developed multiple themes based on iterative review of the raw data using an inductive framework [19] whereby each recommendation was coded and the process was repeated until the coders reached consensus. After the codebook was finalized, the frequency of occurrence of the specific theme (e.g., “increase antidepressant dose”) was tallied to help identify the common types of recommendations made by the psychiatrist.

## Results

Over a six month period, the psychiatrist made recommendations for 192 patients, of which 135 (70%) were female and 57 male (30%). Patient characteristics are listed on Table 1. The participants ranged from age 6 to 77. The total number of recommendations was 214. Because all new and high-priority follow-up patients were discussed, the total number of recommendations exceeded the number of patients. It was most common for the psychiatrist to recommend reevaluations of the primary mental health diagnosis. Often, the psychiatrist suggested that a nonspecific diagnosis (such as “other anxiety disorder”) be refined to a more specific diagnosis (such as “generalized anxiety disorder”). Other times, the psychiatrist suspected depression based on the patient’s presenting concerns, and recommended that the clinicians rule it out. Most recommendations to rule out other medical illness were for the PCPs to exclude hypothyroidism by ordering a thyroid functioning test before diagnosing depression. The vast majority of recommendations related to optimization of treatment, either with adjustment of medication or augmentation with counselling or both. A very common recommendation was to focus on or add counselling or psychosocial support to the treatment plan. This was often recommended for patients with mild to moderate depression who either did not need to take medications or already had their medications optimized. A third common recommendation was to increase the antidepressant dose. For example, patients with moderate to severe depression were receiving 25 mg amitriptyline and the psychiatrist encouraged the PCPs to increase this to a therapeutic dose. In addition, patients who were not improving (e.g., little or no change in PHQ-9 scores over 1.5 months) despite receiving medications (e.g., fluoxetine 20 mg), were recommended to use a higher dose. Another common recommendation was to discontinue medications for patients who were not prescribed appropriate medications (e.g., a patient with psychotic symptoms is prescribed clonazepam or amitriptyline instead of antipsychotics). Only 16% of all patients received recommendations, as the rest were either follow-up patients who already had optimal regimens or new patients where the treatment team used evidence-based practices and the psychiatrist determined that no changes were required. Despite this, only three recommendations were affirmative i.e. the psychiatrist agreed with the local clinical team’s assessments. All the recommendations themes and their frequencies are listed in Table 2.

**Table 1:** Characteristics of patients who received a recommendation from the psychiatrist during panel reviews

Characteristic of participants	*n (%)*
Number of patients receiving recommendations	192
Gender	
Female	135 (70.3%)
Male	57 (29.6%)
Median age	35.5
Diagnosis	
Primary depression	72 (37.5%)
Co-morbid depression	52 (27.1%)
Co-morbid anxiety	36 (18.8%)
Primary anxiety	29 (15.1%)
Other	20 (10.4%)
Primary psychotic disorders	5 (2.6%)

**Table 2:** Frequency of recommendations made by the psychiatrist to improve mental healthcare delivered by primary care providers

Recommendations	Frequency n (% of total recommendations)
Clarification of Psychiatric Diagnosis	55 (25.7%)
Revisit primary mental health diagnosis	34 (15.8%)
Obtain more information from the patients/family	10 (4.6%)
Rule out other medical illnesses (physical)	11 (5.1%)
Treatment optimization	111 (51.8%)
Add or increase focus on counselling and psychosocial support	20 (9.3%)
Increase antidepressants	20 (9.3%)
Discontinue inappropriate medication(s)	12 (5.6%)
Manage sleep problems using non-pharmacological strategies	12 (5.6%)
Add “as-needed” medications	11 (5.1%)
Decrease medication dose	11 (5.1%)
Manage sleep problems using medication	9 (4.2%)
Add propranolol	8 (3.7%)
Add antipsychotic	8 (3.7%)
Other	48 (22.4%)
Total	214

^a^See Additional file 1 for the breakdown of themes under “other” category

## Discussion

Task-sharing, the strategy to utilize non-specialists in delivering healthcare that is typically provided by a specialist, can improve access to mental healthcare [20]. Studies have shown task sharing to be effective in improving outcomes for numerous mental illnesses, including depression, PTSD, and alcohol use disorders [21]. Although there are many advantages of task sharing, there are some significant challenges. Task-sharing places the PCPs at the frontline of providing mental healthcare but as our previous study found, medical school curricula in Nepal substantially deprioritizes mental health [12]. As PCPs will be newly trained on fundamentals of evidence-based mental health practices, there is a high likelihood that they will be prone to making errors. Identifying these errors can help develop training programs and guide new programs to anticipate and resolve challenges. The results of our study reinforce the findings that CoCM can help maintain the quality of mental health services [22].

“Other anxiety” was a common and non-specific diagnosis that was used whenever the patient presented with any worries, regardless of other symptoms, such as depressed mood. Making the correct mental health diagnosis is a challenge in task-sharing programs and our study found this was the most common concern stated by the psychiatrist.

Due to severe shortage of behavioral health professionals, access to evidence-based counselling is severely limited in LMICs. PCPs, in turn, are not used to incorporating non-pharmacological treatments. The World Health Organization’s mental health protocol, mhGAP, does include counselling but given the lack of access, PCPs often rely primarily on medications, even for mild illnesses [23].

The recommendation to start medication at a lower dose was made when patients with chronic psychosis were initiated on 10 mg haloperidol. The recommendation to use a higher dose was mainly directed at increasing amitriptyline beyond the initiation dose of 25 mg. Few patients were prescribed inappropriate medications, including antidepressants for a history that had high suspicion for mania, and using intramuscular haloperidol when oral tablets may have sufficed.

Only 11% of all patients received written recommendations, implying that the psychiatrist was satisfied with the diagnoses and treatment plans for the vast majority of patients. However, only three documented recommendations were affirmative or positive. Affirmative recommendations may help improve PCPs’ morale and can also help to reduce the association between a recommendation and a criticism. If the psychiatrist included positive feedback as part of these recommendations, PCPs may welcome such comments and that may further boost morale. Another important result is that only one recommendation was to assess for medication adherence. Given the high rates of non-adherence in mental health treatment, this is likely an indication for the treatment team to consider adherence issues in greater frequency.

## Conclusion

Here, we identified major areas in which a consulting psychiatrist provided recommendations to rural PCPs and counselors using CoCM. The results have led the CoCM team to focus on the top four most commonly discussed recommendations. While this study represents only one clinical site, our data provide a foundation for a larger study of the functioning, challenges, and utility of CoCM models in rural areas using remote psychiatrists.

Our findings show that the most commonly received recommendations were to revisit primary mental health diagnosis and to focus on counselling Both these findings are consistent with published research about prevalence of diagnostic errors in mental health [24] and underuse of non-pharmacological interventions like counselling in treating mental illness. The process of reviewing recommendations received in CoCM can help guide future trainings, continuous medical education curricula, and further research while also seeking to improve the quality and effectiveness of mental healthcare delivery in similar settings worldwide.

## Supplementary information

**Additional file 1.** Frequency of recommendations made by the psychiatrist to improve mental healthcare delivered by primary care providers. Additional file 1 lists frequency of recommendation including breakdown of themes under “other” category.

## Data Availability

The datasets used and/or analyzed during the current study are available from the corresponding author on reasonable request.

## Abbreviations

CoCM: Collaborative care model
EHR: Electronic health record
LMIC: Low- and middle-income countries
mhGAP: Mental health gap action program
PCP: Primary care provider
PHQ-9: Patient health questionnaire
PTSD: Post-traumatic stress disorder
