# Supplementary material for: Treatment recommendations made by a consultant psychiatrist to improve the quality of care in a collaborative mental health intervention in rural Nepal
Source: BMC Psychiatry. 2020 Feb 5;20:46. doi: 10.1186/s12888-020-2464-1 (PMC7003398; doi:10.1186/s12888-020-2464-1)
Supplement: Supplementary file 1 — Additional file 1. Frequency of recommendations made by the psychiatrist to improve mental healthcare delivered by primary care providers. Additional file 1 lists frequency of recommendation including breakdown of themes under “other” category. [file 12888_2020_2464_MOESM1_ESM.docx]

| *Recommendations* | *Frequency n (% of total recommendations)* |
| --- | --- |
| Clarification of Psychiatric Diagnosis | 55 (25.7%) |
| Revisit primary mental health diagnosis | 34 (15.8%) |
| Obtain more information from the patients/family | 10 (4.6%) |
| Rule out other medical illnesses (physical) | 11 (5.1%) |
| Treatment optimization | 111 (51.8%) |
| Add or increase focus on counselling and psychosocial support | 20 (9.3%) |
| Increase antidepressants | 20 (9.3%) |
| Discontinue inappropriate medication(s) | 12 (5.6%) |
| Manage sleep problems using non-pharmacological strategies | 12 (5.6%) |
| Add “as-needed” medications | 11 (5.1%) |
| Decrease medication dose | 11 (5.1%) |
| Manage sleep problems using medication | 9 (4.2%) |
| Add propranolol | 8 (3.7%) |
| Add antipsychotic | 8 (3.7%) |
| Increase meds dose (other than antidepressants and antipsychotics) | 6(2.8%) |
| Assess medication side effect | 6(2.8%) |
| Start medication in lower dose | 5(2.3%) |
| Avoid medication in mild illness | 4(1.8%) |
| Refer patients for higher level care | 4(1.8%) |
| Avoid long term benzodiazepine use | 4(1.8%) |
| Stop medication (treatment completed) | 3 (1.4%) |
| Add antidepressants | 3 (1.4%) |
| Affirmative recommendations | 3 (1.4%) |
| Consider co-morbid mental illness | 3 (1.4%) |
| Switch from amitriptyline to fluoxetine | 2(0.9%) |
| Increase antipsychotic dose | 2(0.9%) |
| Provide psychoeducation about risk of medication | 2(0.9%) |
| Assess for medical adherence | 1(0.4%) |
| Total | 214 (100%) |

Additional file 1 lists frequency of recommendations including breakdown of themes under “other” category
